# Understanding how social norms affect modern contraceptive use

**DOI:** 10.1186/s12889-021-11110-2

**Published:** 2021-06-04

**Authors:** Sohail Agha, Brooks Morgan, Helena Archer, Shadae Paul, Joseph B. Babigumira, Brandon L. Guthrie

**Affiliations:** 1 The Bill & Melinda Gates Foundation, Seattle, USA; 2 The University of Washington, Seattle, USA

## Abstract

**Background:**

An aim of this study is to introduce a practitioner-friendly behavior model. Few theories of health behavior explicitly take the effect of social norms on behavior into account. Generally, theories that do take social norms into account assume that the effect of social norms on behavior operates through motivation. We use the Fogg Behavior Model (FBM), a behavior model that is new to public health, to explore whether social norms are associated with modern contraceptive use among Nigerian women, and whether they affect behavior through motivation or through ability. In other words, do social norms that discourage contraception lower women’s motivation to use contraception or do they lower women’s ability to use contraception.

**Methods:**

This study uses data from a cross-sectional household survey of Nigerian women, ages 14–24. The survey collected data on socio-economic and demographic characteristics of women, whether they were sexually experienced, and whether they used contraception. Modern contraceptive use was the outcome of interest for the study. The survey also collected data on social norms around premarital sex and contraceptive use. Multivariate logistic regression was used for the analysis.

**Results:**

After adjusting for a range of socio-economic and demographic variables, we found that social norms that discourage contraception had a statistically significant negative association with contraceptive use (aOR = 0.90, *p* < 0.001). The analysis found that the negative association between social norms and contraceptive use remained statistically significant after controlling for motivation but did not remain statistically significant after controlling for ability.

**Conclusion:**

These findings suggest that social norms may affect contraceptive use in Nigeria through ability rather than motivation. In terms of programmatic implications, these finding suggest that public health interventions may be able to counter the negative effects of social norms that discourage contraceptive use by increasing women’s ability to practice contraception.

## Background

Adolescent pregnancy is a major reason why girls drop out of school and into poverty [1]. This is pertinent to Nigeria where more than half the population is under 24 years old [2]. A Nigerian study among female students 15–24 found that 68% had had unwanted pregnancy and 64% had had an induced abortion. While all students were aware of contraception, only 25% had ever used it [3]. Studies show that perceived community disapproval of contraceptive use is one of the most important reasons for non-use of modern contraception among 15–24 year old Nigerian women [1, 3].

In recent years, there has been an upsurge of interest in social norms and their effects on contraceptive use behavior [4–7]. The interest in social norms comes from a growing recognition that development efforts have largely focused on nonsocial components [8] such as ensuring the availability of immunizations at health facilities or ensuring that trained providers are available to provide quality labor and birth services. While there is no denying the importance of these nonsocial components, there is increasing recognition among donors and practitioners that the impact of social factors has been vastly underestimated [9]. This comes from the realization that, for example, immunization rates are far below expected levels in contexts where vaccines are widely available or that the uptake of contraceptive services is well below what would be expected based on the unmet need for contraception.

The growing interest among donors and practitioners in the role of social factors in the human environment, and social norms, is welcome. At the same time, it is critical to recognize an important limitation in the social and behavioral sciences: while there are more than 80 theories of health behavior [10], few theories have taken normative change into account. We believe that, for donor interest to translate into an allocation of resources that systematically takes normative factors into account, it is important to locate social norms within a practical model of behavior change - one that is accessible to a broad range of stakeholders. Such a model could play a central role in helping donors and practitioners develop a consistent understanding of the effects of social norms on behavior. Alternatively, the absence of such a model may impede public health practitioners from implementing normative interventions within a behavior change framework.

Most of the commonly used theories of health behavior such as the Health Belief Model, the Transtheoretical Model, and the Social Ecological Model do not explicitly address social norms. The Theory of Reasoned Action (TRA), and its extension the Theory of Planned Behavior (TPB), are perhaps the only commonly used theories that include an explicit consideration of social norms as drivers of behavior. The TRA explains an individual’s behavior in terms of the underlying motivation to perform an action. It considers the intention to perform an action to be, in part, determined by social norms or the “perceived social pressure” to perform that action. The TPB differs from the TRA only in that it adds perceived behavioral control as an additional determinant of motivation [11].

Another theory of human behavior used to design health and development interventions is the Social Cognitive Theory (SCT). The SCT considers the concept of self-efficacy, or a person’s ability to persist with an action despite challenges, as critical to the adoption of a behavior [12, 13]. The SCT is based on the idea that a person learns from observing the behavior of others. Modeling of behavior is one of the strongest influences on a person’s judgement of their self-efficacy. Individual ability is important in the SCT, with the function of health interventions being to deliberately increase a person’s confidence in their capacity to adopt and sustain a behavior. While social norms are not measured explicitly in the SCT, Bandura considers their function to be to encourage or discourage a new behavior by conveying behavioral standards according to which an individual evaluates her self-efficacy to adopt a behavior [13]. For example, studies have shown that when people perceive substance use to be high and approved by others, their confidence in their ability to resist the temptation to use marijuana declines. Thus, social norms that encourage marijuana use reduce a young adult’s ability to refuse marijuana [14].

Social Norms Theory (SNT) itself, a theory which is not used frequently to design public health interventions in low-and-middle-income countries, highlights the role of peer influences in individual decision-making. This theory makes the distinction between “perceived” and “real” norms. According to this theory, peer influences, affected by perceived rather than real norms, become the basis for misperceptions that lead to unhealthy behaviors such as excessive alcohol consumption. Interventions that correct these misperceptions are expected to lead to a reduction in problematic behaviors [15]. The SNT highlights the importance of presenting correct information about peer group norms to drive changes in behavior but does not directly link social norms to either motivation or ability. It assumes a direct effect of social norms on behavior.

Given the limited attention to social norms in health behavior change theories, it is perhaps not surprising that there has been little exploration of whether social norms affect behavior through motivation or through ability. This investigation is important because of its implications for the design of public health interventions: the design of an intervention to increase motivation may be quite different from that of an intervention to increase ability. For example, an intervention may increase a young woman’s motivation to practice contraception by increasing community dialogue about the negative consequences of unwanted teenage pregnancy, while another intervention may increase her ability to practice contraception by making contraceptives widely available in the community and ensuring that she knows how to access contraceptive services. In this study, we test the following hypotheses:

H1: Social norms that discourage premarital sex and contraceptive use have no effect on modern contraceptive use.

H2: The effect of social norms on modern contraceptive use does not operate through women’s motivation to practice contraception.

H3: The effect of social norms on modern contraceptive use does not operate through women’s ability to practice contraception.

In addition to looking at social norms around contraceptive use, we consider the influence of social norms that discourage premarital sex because the decision to become sexually active and the decision to use contraception are joint decisions [16]. We use the Fogg Behavior Model (FBM) to explore the mechanism through which social norms affect contraceptive use. The FBM states that behavior happens when motivation, ability and a prompt occur in the same moment. In other words, motivation and ability must both be present for behavior to occur.

**Fig. 1:**
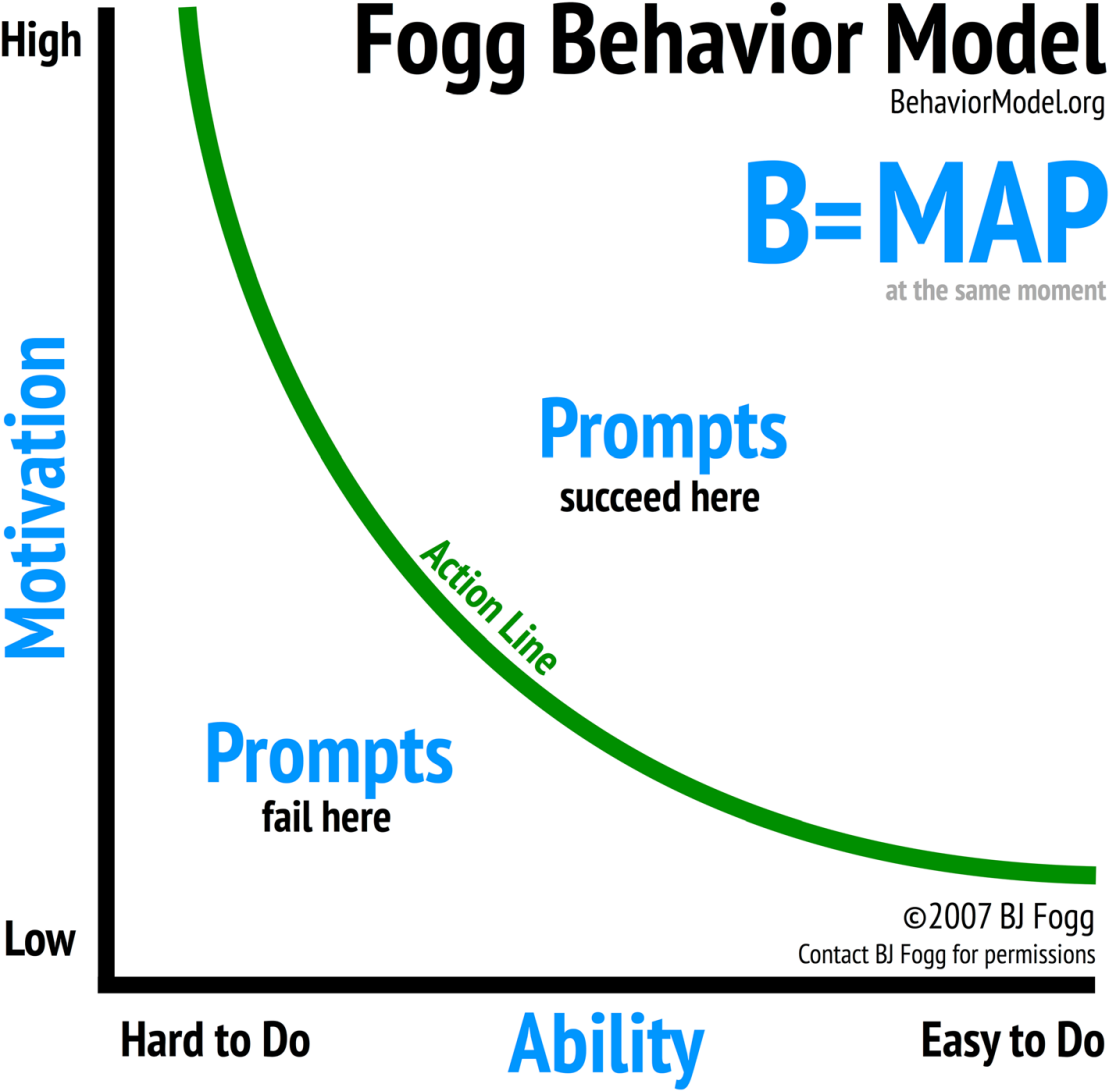
Fogg behavior model

The FBM can be visualized in two dimensions, with motivation on the y-axis and ability on the x-axis, as shown in Fig. 1. For a specific behavior, motivation can range from high to low, as can ability. The FBM states that a person with high motivation and high ability will adopt a behavior when prompted. By contrast, a person with low motivation and low ability will not adopt a behavior when prompted [17]. Using panel data from household surveys, a recent study found a significantly higher odds of condom use among men with high motivation and high ability compared to men with low motivation and low ability. Men with high motivation and high ability also had a significantly higher odds of condom use compared to men who had high levels of either motivation or ability but not both [18], as suggested by the FBM.

An advantage of using the FBM over other practitioner-friendly models of behavior is that it provides clear and specific definitions of the components of motivation and ability. Because the FBM includes both motivation and ability as critical elements needed for behavior to change, it allows us to explore whether the relationship between social norms and behavior is reliant on motivation or on ability.

## Methods

### Survey design and data collection

This study used data from a cross-sectional household survey conducted in Nigeria. The PMA2020 Nigeria sample frame in Lagos, Kaduna, and Kano was used to draw the sample for this survey [19]. A representative number of geographic clusters (“enumeration areas”) were sampled in these states. Interviews were conducted in a total of 62 clusters (18 in Kaduna, 15 in Kano and 29 in Lagos). Age and the capacity to consent to survey participation were the eligibility criteria. Systematic random sampling was used to select households. Within households, women ages 14–24 were eligible for the study. The training of interviewers was conducted in Abuja, the capital, between February 13th and 17th, 2018. Interviewers were selected from the particular state where the survey was conducted and were fluent in local languages. Training was conducted using paper versions of the instrument before android phones were used for survey data collection.

Two pilots were conducted in Niger state before the instrument was finalized. The data collection was conducted between February 19 and March 4, 2018. The data was used to construct variables used in our analysis, including variables measuring social norms, motivation, ability, and modern contraceptive use.

Spatial data was used to ensure that each interviewer went to the location they were assigned. The Open Data Kit software captured GPS co-ordinates verified using Google Earth. This allowed survey managers to determine whether the location in which the interview was conducted was correct. A performance dashboard was used to monitor interviewer error, the time taken for each interview, and the number of interviews completed per day by an interviewer.

Interviews were completed with 1916 women, ages 14–24, out of a total of 2051 eligible women, a participation rate of 93%. Households were listed and mapped in each enumeration area. In the Southern state, Lagos, 604 women were interviewed and in the Northern states of Kaduna and Kano 651 and 646 women were interviewed, respectively. All women were asked: “Are you currently married or living together with a man as if married?” Women who were not currently married were asked: “Do you currently have a boyfriend?” A total of 628 women reported being married, living together with a man as if married or having a boyfriend. Being married or living with a man was an inclusion criterion for the analysis.

Data on the outcome, modern contraceptive use, was missing for 10 women. Women with missing data were dropped from the analysis. Further analysis was conducted with data from 618 women who were married, living with a man as if married or who had a boyfriend. Current use of modern contraception was 27% among these women. Condoms were the primary modern method being used, with 64% of current modern method users reporting the use of condoms.

### Social norms

Researchers from a range of disciplines including sociology, economics, gender studies and psychology have been interested in the influence of social norms on behavior. A social norm is what people in a group consider to be a typical and appropriate behavior in a particular context [20]. It is held in place by reciprocal expectations of members of an individual’s reference group [8]. Cialdini [21] provided clarity to the concept of social norms by distinguishing two key types of social norms: a descriptive norm is what a person believes that relevant others around her do; an injunctive norm is what a person believes that relevant others expect her to do.

In other words, a descriptive norm is a person’s perception of how widespread a specific behavior is while an injunctive norm is a person’s perception of whether that behavior is socially approved. For example, the descriptive norm around premarital sex among young women in one context may be that unmarried women do not have sex before marriage. The injunctive norm in such a context may be that women who have sex before marriage are considered promiscuous.

We classified normative questions available in the Nigeria survey into two categories: descriptive or injunctive. Bivariate analysis showed that variables based on 7 of the 19 survey questions that reflected social norms had a statistically significant relationship with modern contraceptive use. These 7 variables were retained for further analysis. Social norms variables were recoded so that a higher score indicated a more unfavorable view of premarital sex and contraceptive use.

Table 1 shows survey questions measuring descriptive and injunctive norms related to premarital sex and contraceptive use. Questions measuring descriptive norms include “most of my friends are having sex before marriage” and “most adolescent girls talk to their boyfriends about contraceptives”. Questions measuring injunctive norms include “most of my friends think that adolescents who do not have sex before marriage are old-fashioned” and “a woman who uses contraception without her husband’s knowledge should be punished”. Based on the addition of scores on individual variables, each survey respondent received a social norms score. Social norms questions were measured on a 4-point scale. The social norms score ranged from 7 to 28. The Cronbach alpha for the social norms scale was 0.645.

**Table 1:** Descriptive and Injunctive Norms around Sexual Initiation and Contraceptive Use

NORMS	Questions from survey used in analysis
	*Please tell me if you strongly agree, somewhat agree, somewhat disagree or don’t know.*
**Descriptive**	329. Most of my friends are having sex before marriage.
**Descriptive**	331. Most of my friends think female adolescents do not have to maintain their virginity.
**Descriptive**	557. Most adolescent girls talk to their partners/boyfriends about contraceptives.
**Injunctive**	330. Most of my friends think it is cool to have sex at my age.
**Injunctive**	332. Most of my friends think that adolescents who do not have sex before marriage are old-fashioned.
**Injunctive**	554. A woman who uses contraception without her husband’s knowledge should be punished.
**Injunctive**	556. Most of my friends would approve of my using contraceptives.

### Operationalization of the Fogg behavior model: *Motivation and Ability*

To operationalize the FBM, we identified survey items that were consistent with Fogg’s definition of motivation and ability. Concurrently, we reviewed the broader literature and identified motivation and ability factors that predict contraceptive use [22–32]. We then classified questions in the survey instrument into motivation or ability categories.

Thirty-two survey questions were identified that reflected motivation and 25 that reflected ability. The behavior of interest was a woman’s self-reported use of modern contraception. Bivariate analysis showed that variables based on 9 of the 32 questions representing motivation and 7 of the 25 questions representing ability did not have a statistically significant relationship with modern contraceptive use (at *p* < 0.05). These 16 variables were not retained for further analysis, leaving 23 variables to represent motivation and 18 variables to represent ability.

Table 2 shows the 23 survey questions measuring motivation that were used in our analysis. Fogg identifies 3 core motivators: sensation (characterized by pleasure and pain), anticipation (characterized by hope and fear) and belonging (characterized by acceptance and rejection). To measure sensation, respondents were asked about the extent to which they agreed or disagreed with statements such as “condoms ruin the mood” or “contraceptives reduce a man’s sexual urge”. To measure anticipation, women were asked about their agreement or disagreement with statements such as “condoms prevent pregnancy” or “condoms have holes that allow HIV to pass through them”. Belonging was measured by asking respondents to agree or disagree with statements such as “adolescent girls who have sex before marriage should feel ashamed” or “women who use contraceptives may become promiscuous”.

**Table 2:** Motivation questions that correspond to the Fogg Behavior Model

MOTIVATION COMPONENT	Questions from survey used in the analysis
	*Please tell me if you strongly agree, somewhat agree, somewhat disagree, strongly disagree or don’t know.*
**Sensation**	407. Sex is unnatural with condoms.
**Sensation**	408. Condoms ruin the mood.
**Sensation**	545. Contraceptives reduce a woman’s sexual urge.
**Sensation**	546. Contraceptives reduce a man’s sexual urge.
**Anticipation**	412. Condoms prevent pregnancy.
**Anticipation**	414. Condoms have holes that allow HIV to pass through them.
**Anticipation**	547. Contraceptives can cause cancer.
**Anticipation**	549. Contraceptives are dangerous to your health.
**Anticipation**	420a. How motivated or unmotivated are you to use condoms with your partner? Very motivated, somewhat motivated, unmotivated, very unmotivated or don’t know.
**Anticipation**	535a. How motivated or unmotivated are you to discuss contraception with your partner? Very motivated, somewhat motivated, unmotivated, very unmotivated or don’t know.
**Anticipation**	566. How motivated on unmotivated are you to use contraception? Very motivated, somewhat motivated, unmotivated, very unmotivated or don’t know.
**Anticipation**	536. Do you intend to talk to your partner about contraception in the next three months?
	*Please tell me if you strongly agree, somewhat agree, somewhat disagree, strongly disagree or don’t know.*
**Belonging**	319. It is against your values to have sexual intercourse before marriage.
**Belonging**	322. Adolescent girls who get pregnant before marriage should feel ashamed.
**Belonging**	325. Adolescents should have sex before marriage to see if they are suited to each other.
**Belonging**	411. Condom use means that a person is promiscuous.
**Belonging**	550. Women who use contraceptives may become promiscuous.
**Belonging**	544. Use of some contraceptives can make a woman permanently infertile.
**Belonging**	552. The husband should be the one to decide whether the couple should use a method of contraception.
**Belonging**	317a. On a scale of 1–7 please tell me how having sex makes a person cool. 1 is not cool and 7 is cool.
**Belonging**	317c. On a scale of 1–7 please tell me how having sex makes a person sexy. 1 is not sexy and 7 is sexy.
**Belonging**	317d. On a scale of 1–7 please tell me how having sex makes a person respected. 1 is not respected and 7 is respected.
**Belonging**	530. Have you and your partner ever discussed the number of children you would like to have?

Eighteen out of 23 variables measuring three core motivators were measured on a 4-point scale going from strongly agree to strongly disagree, with a “don’t know” option provided. Two of the 23 variable which fell under motivation had binary response options: “have you and your partner ever discussed the number of children you would like to have” and “do you intend to talk to your partner about contraception in the next 3 months”. Each of these two questions had a maximum score of 1. The remaining 3 questions had a maximum score of 7: “on a scale of 1-7 please tell me how having sex makes a person cool”. Motivation variables were recoded so that their values went from low to high. Based on the addition of scores on individual variables, each survey respondent received a motivation score. Motivation scores ranged from 26 to 87. The Cronbach alpha for the motivation scale was 0.797.

Table 3 shows the 18 survey questions measuring ability. Fogg identifies 5 core elements within ability: time, money, mental effort, physical effort, and routine. The survey instrument did not have measures of time, money, or physical effort. Ability questions available in the instrument primarily reflected mental effort. Respondents were asked to agree or disagree with statements such as “using condoms during sexual intercourse is embarrassing” or “how easy or difficult would it be for you to use condoms with a sexual partner”. One question was available on routine: “do you carry condoms with you?”

**Table 3:** Ability questions that correspond to the Fogg Behavior Model

ABILITY COMPONENT	Questions from survey used in the analysis
	*Please tell me if you strongly agree, somewhat agree, somewhat disagree, strongly disagree or don’t know.*
**Mental effort**	409. Using condoms during sexual intercourse is wise.
**Mental effort**	410. Using condoms during sexual intercourse is embarrassing.
	*Please tell me how easy or difficult it would be to do each of the following. Would it be very easy, somewhat easy, somewhat difficult, very difficult or you don’t know.*
**Mental effort**	420. How easy or difficult would it be for you to use condoms with a sexual partner?
**Mental effort**	421. How easy or difficult would it be for you to discuss condoms with a sexual partner?
**Mental effort**	422. How easy or difficult would it be for you to discuss condoms with your parents?
**Mental effort**	565. How easy or difficult is it for you to use contraception?
	*Please tell me how confident you would feel, extremely confident, somewhat confident, somewhat uncertain, extremely uncertain or you don’t know.*
**Mental effort**	423. How confident are you that you could get a condom if you wanted one?
**Mental effort**	424. How confident are you that you could have a condom with you when you needed it, that is if you decided to have sex?
**Mental effort**	425. How confident are you that you could use a condom correctly?
**Mental effort**	426. Imagine that you are having sex with someone you just met, and you feel it is important to use condoms. How confident are you that you could tell that person you want to use condoms?
**Mental effort**	427. Imagine that your partner uses birth control pills to prevent pregnancy. You want to use condoms to avoid getting an STD or HIV. How confident are you that you could convince your partner to also use condoms?
**Mental effort**	563. How confident are you that you could convince your partner to use a method of contraception?
**Mental effort**	564. How confident are you that you could use a method of contraception even if your partner doesn’t want to?
	*Now I am going to ask you about the likelihood of some events. Please tell me if you would be extremely unlikely, somewhat unlikely, somewhat likely, extremely likely or don’t know.*
**Mental effort**	429. How likely is it that your partner would like it if you had a condom with you?
**Mental effort**	430. During the next 3 months, how likely is it that you will try to persuade your partner to use condoms every time you have sex?
**Mental effort**	432. During the next 3 months, how likely is it that you will always have a condom with you?
**Mental effort**	517. Do you know of a place where you can obtain a method of contraception?
**Routine**	404. Do you carry condoms with you?

Sixteen out of 18 ability variables were measured on a 4-point scale. Two of the 18 ability variables had binary response options: “do you carry condoms with you” and “do you know of a place where you can obtain a method of contraception”. Each of these two questions had a maximum score of 1. Ability variables were also recoded so that their values went from low to high ability. Based on the addition of scores on individual variables, each survey respondent received an ability score. Ability scores ranged from 16 to 66. The Cronbach alpha for the ability scale was 0.883. For both motivation and ability variables, missing values were recoded to the mean. For most variables, missing values were less than 5%. Both ability and motivation were correlated with social norms. The correlation between the social norms and the motivation variable was − 0.31 (*p* = 0.000). The correlation between the social norms and the ability variable was − 0.43 (*p* = 0.000).

### The outcome variable: *Modern Contraceptive Use*

Modern contraceptive use was the outcome of interest for this study. Respondents were asked “Are you currently doing something or using any method to avoid getting pregnant?” Women who responded in the affirmative were asked about the method of contraception they were currently using. Women were asked about the following modern methods: the IUD, the injectable, the implant, the oral contraceptive, the male condom, the female condom, the diaphragm, the foam or jelly, and the Standard Days. Women who were currently using any of these methods were classified as modern contraceptive method users. The outcome variable was binary. Women were also asked about the use of traditional methods, including lactation amenorrhea, withdrawal, and rhythm. Traditional methods are not included in our definition of modern method.

### Statistical analysis

Frequency distributions of socio-economic and demographic characteristics of the sample were calculated. Cross tabulations were conducted to show the relationships between categorical variables and the outcome of interest, modern contraceptive use. Chi-square tests of independence were conducted at the bivariate level. ANOVA was used to show the relationships between contraceptive use and social norms, motivation, and ability.

We explored the relationship between social norms and contraceptive use in a multivariate framework. We used a logistic regression model [33] for the analysis, with the log odds of the outcome modelled against a linear combination of explanatory variables. The clustering of observations (i.e. clustering of women within localities) was taken into account by use of the STATA cluster command [33].

### Ethics review

For Nigeria, Tulane University Biomedical IRB approval was received – IRB reference number 2017–6388. Local IRB approval was obtained from the Nigerian National Health Research Committee (NHREC) – NHREC/01/01/2007–24/01/2018. All protocols were carried out in accordance with relevant guidelines and regulations. Informed consent was obtained from all study participants.

Research staff from the Centre for Research, Evaluation Resources, and Development (CRERD) obtained written consent from all eligible participants prior to conducting any interviews. During household visits, local research staff explained the purpose of the research study, the survey content, the survey procedures, the risks and benefits of participation, compensation (none) and interviewer rights and autonomy. Written informed consent form was obtained from youth aged 18–24 years. Parental consent and youth assent were solicited from youth aged 14–17 years. Married youth were considered emancipated and able to consent for themselves.

## Results

### Socio-demographic characteristics of women and modern contraceptive use

Column 1 of Table 4 shows the frequency distributions of characteristics of women in the sample. About one-third of women were from the South and two-thirds were from the North. Slightly more than a quarter of respondents were adolescents, ages 14–19. Nearly half of women in the sample did not have a child. About 64% of women were married. Nearly two-thirds of women had secondary education and 14% had higher than secondary education.

**Table 4:** Women’s characteristics and modern contraceptive use

	(1) Women’s characteristics (*n* = 618)	Modern Contraceptive Use		(4) ^1^*p*-value
		(2) Not using modern contraception	(3) Currently using modern contraception	
**Region**				
South	34.1%	65.9%	34.1%	
North	65.9%	76.4%	23.6%	0.004
**Age**				
14–19	26.2%	77.8%	22.2%	
20–24	73.4%	71.1%	28.9%	0.098
**Number of children**				
1–3	52.1%	78.6%	21.4%	
None	47.9%	66.6%	33.4%	0.001
**Marital status**				
Married	64.1%	81.8%	18.9%	
Boyfriend	35.9%	58.1%	41.9%	0.000
**Education**				
None or primary	19.4%	80.0%	20.0%	
Secondary	66.7%	75.0%	25.0%	
Higher than secondary	13.9%	52.3%	47.7%	0.000
**Wealth**				
Second to fifth quintiles	79.9%	69.8%	30.2%	
First quintile/Poorest	20.1%	84.7%	15.3%	0.001
**Mean social norms score**	18.0	18.8	16.0	0.000
**Mean motivation score**	60.6	58.0	67.7	0.000
**Mean ability score**	38.0	34.8	46.5	0.000

^1^Chi-square tests were used for categorical variables and Anova was conducted for numerical variables

Columns 2 and 3 of Table 4 show reported contraceptive use among women in the sample. Column 4 shows *p*-values associated with statistical tests conducted at the bivariate level. Contraceptive use was higher in the South compared to the North: 34% of women in the South compared to 24% in the North used a modern contraceptive method (*p* = 0.004). There was no statistically significant difference in contraceptive use by age. Women without a child were significantly more likely to use a contraceptive: 33% of women without a child reported contraceptive use compared with 21% of women who had one or more children (*p* = 0.001). Women with boyfriends were more likely to use a contraceptive method compared with women who were married (42% versus 19%, *p* = 0.000). Women with higher than secondary education were more likely to use a contraceptive method (48%) compared with women with secondary (25%) or lower than secondary education (20%)(*p* = 0.000). The poorest women, women in the first quintile, were less likely to use a contraceptive method (15%) than other women (30%)(*p* = 0.001).

Women who were not currently using a modern contraceptive method had a higher score on the variable measuring social norms that discourage premarital sex and modern contraception (18.8 versus 16.0, *p* = 0.000). Women who were currently using contraception had higher scores on variables measuring motivation (67.7 versus 58.0, *p* = 0.000) and ability (46.5 versus 34.8, *p* = 0.000) than women who were not.

### Motivation, ability and modern contraceptive use: regression analysis

We introduced variables in stages, using an approach similar to path analysis, where the variance explained by variables introduced later in the model helps explain the variance of variables introduced earlier in the model. Four models were used. The first model shows the odds of contraceptive use by socio-economic and demographic characteristics. The second model adds a variable measuring social norms that discourage contraceptive use. This model explains whether there is a relationship between social norms and contraceptive use, independent of other factors. The third model adds a variable measuring motivation to Model 2. The fourth model adds a variable measuring ability to Model 2. Models 3 and 4 help illustrate whether the effects of social norms are associated with motivation or with ability. A *p*-value of 0.05 was used as a threshold for tests of statistical significance conducted in this study. The analyses were conducted in STATA Version 15.

Logistic regression analysis, shown in Table 5, reveals significant associations between modern contraceptive use and socio-economic and demographic characteristics, social norms, motivation, and ability. Model 1, in Table 5, shows the relationship between socio-economic and demographic characteristics and contraceptive use. After adjusting for age, the number of children, marital status, education, and wealth, there was no statistically significant difference in contraceptive use by region. Women ages 20–24 were significantly more likely to use a modern contraceptive method compared with women 14–19 (aOR = 1.68, *p* < 0.05). Women with a boyfriend were more likely to use contraception than married women (aOR = 3.42, *p* < 0.001). Women in the poorest quintile were significantly less likely to report using contraception (aOR = 0.49, *p* < 0.01). Once wealth and other variables were included in the model, the number of children and education were not associated with contraceptive use.

**Table 5:** Logistic regression showing the adjusted odds of modern contraceptive use among young Nigerian women

	Model 1 aOR (95% CI)	Model 2 aOR (95% CI)	Model 3 aOR (95% CI)	Model 4 aOR (95% CI)
**Region**				
South	1.00	1.00	1.00	1.00
North	0.93 (0.64, 1.33)	1.21 (0.82, 1.79)	1.62* (1.02, 2.58)	1.10 (0.72, 1.67)
**Age**				
14–19	1.00	1.00	1.00	1.00
20–24	1.68* (1.02,2.75)	1.62 (0.99,2.64)	1.37 (0.84,2.25)	1.27 (0.78,2.07)
**Number of children**				
1–3	1.00	1.00	1.00	1.00
None	0.87 (0.53,1.44)	0.89 (0.55,1.46)	0.98 (0.58,1.65)	0.97 (0.56,1.71)
**Marital status**				
Married	1.00	1.00	1.00	1.00
Boyfriend	3.42***(2.02,5.77)	2.82***(1.71,4.65)	2.97***(1.76,4.99)	1.76*(1.01,3.06)
**Education**				
None or primary	1.00	1.00	1.00	1.00
Secondary	0.79 (0.48,1.31)	0.73 (0.43,1.24)	0.67 (0.40,1.11)	0.62 (0.37,1.04)
Higher than secondary	1.63 (0.79,3.37)	1.49 (0.67,3.32)	1.16 (0.54,2.48)	1.02 (0.44,2.39)
**Wealth**				
Second to fifth quintiles	1.00	1.00	1.00	1.00
First quintile/Poorest	0.49** (0.29,0.81)	0.51* (0.29,0.89)	0.52* (0.27,0.97	0.59 (0.33,1.04)
**Social norms scale**	*Not included*	0.90***(0.86,0.95)	0.92**(0.88,0.97)	0.95 (0.90,1.00)
**Motivation scale**	*Not included*	*Not included*	1.07***(1.05,1.09)	*Not included*
**Ability scale**	*Not included*	*Not included*	*Not included*	1.08***(1.06,1.10)
Pseudo R^2^	8.59%	11.31%	18.79%	19.87
Number of women	618	618	618	618

*p < 0.05 **p < 0.01 ***p < 0.001 *aOR* Adjusted Odds Ratios; *95%CI* 95% Confidence Interval

In Model 2, we introduced the variable measuring social norms. Even after adjusting for socio-economic and demographic characteristics, there was a significant negative association between social norms that discourage premarital sex and contraceptive use and the use of contraception. A higher score on the social norms variable was associated with lower contraceptive use (aOR = 0.90, *p* < 0.001).

In Model 3, we added a variable measuring motivation to use contraception to the variables in Model 2. Higher motivation to use contraception was associated with higher contraceptive use (aOR = 1.07, *p* < 0.001). It is important to note that there was no change in the relationship between social norms and modern contraceptive use after motivation was added to the model. This suggests that the effect of social norms on contraceptive use is independent of motivation.

In Model 4, we added a variable measuring ability to use contraception to the variables in Model 2. An increase in the ability to use contraception was associated with higher contraceptive use (aOR = 1.08, *p* < 0.001). We found that the social norms variable was no longer associated with contraceptive use once ability was added to the model. This suggests that the effect of social norms on modern contraceptive use is associated with ability. In other words, social norms that discourage premarital sex and contraceptive use may negatively affect contraceptive use by lowering women’s ability to use contraception.

Earlier (Model 2) we saw the negative association between being poor and modern contraceptive use. This association remained after adjusting for motivation (Model 3) but disappeared once ability was taken into account (Model 4). This is an important finding and suggests that the lower level of contraceptive use among the poorest Nigerian women stems from low ability to use contraception, rather than from low motivation.

## Discussion

We used a model of behavior that is new to public health, the Fogg Behavior Model (FBM). The FBM states that behavior happens when motivation, ability and a prompt happen in the same moment. In the FBM, motivation is conceptualized as stemming from a person’s hopes and fears related to the behavior, the pleasure or pain that they experience from the behavior, and what the behavior means for their sense of belonging. Ability is comprised of practical factors such as time, money, physical effort, as well as factors such as mental effort and routine. Unlike most commonly used theories of behavior used in public health, the FBM considers both motivation and ability to be important drivers of behavior. This provides us with the opportunity to explore whether social norms influence contraceptive use through motivation or through ability.

Consistent with the literature, the study found that social norms that discourage premarital sex and contraceptive use are associated with lower levels of contraceptive use [1, 24, 34–36]. Our findings suggest that the effect of social norms on behavior is associated with ability. This is an important because it suggests that health interventions that increase women’s ability to use contraception may be able to overcome the negative effects of social norms on contraceptive use.

The ability component measured in our study was the mental effort required to adopt contraception. This construct comprised of the embarrassment associated with contraceptive use, the challenge women face in discussing contraceptive use with their partner, their lack of confidence in being able to obtain a contraceptive method when they need it, their lack of confidence in being able to convince their partner to use contraception, as well as not knowing where to access contraceptive services. Indeed, well designed public health interventions that aim to increase contraceptive use do focus on many of these factors. That social norms are associated with ability is a welcome finding because ability is easier to change than motivation [17]. Motivation is less reliable than ability – it comes in waves. While a motivational boost can be useful in initiating a new behavior, it cannot be relied upon for sustaining that behavior [17].

Indeed, whether social norms operate through motivation or ability has important implications for program design. Many public health interventions, such as social marketing interventions, have considerable experience in increasing the ability of target audiences to adopt new behaviors. They do so by providing practical solutions: subsidizing the price of contraceptives, making contraceptives widely available, and enabling couples to overcome the feeling of embarrassment when discussing contraceptive use with their partner [18, 27, 32]. If intervention designers were to assume that social norms influence contraceptive use through ability, the implications for program design may be very different than if they were to assume that social norms influence contraceptive use through motivation.

An important limitation of this study is the use of existing data from a survey which was not specifically designed to collect data on the constructs articulated by the FBM. As a result, several important elements which comprise ability in the FBM (e.g. time, money, physical effort) were not measured in our study. Another limitation of this study is the reliance on self-reported behavior, which may be influenced by social desirability or recall bias [37].

Finally, because we use cross sectional survey data, no causal inference can be drawn from the analysis. Prospective, experimental, studies are needed to identify the mechanism through which social norms influence behavior. Such studies could be conducted on digital platforms used widely used by adolescents and would be extremely useful in informing future intervention design.

We cannot generalize from a study focused on a specific behavior (modern contraceptive use) whether social norms may influence ability for other behaviors as well. This is an empirical question that should be answered through the collection and analysis of data on social norms related to specific behaviors. The increasing interest in the influence of social norms on behavior among public health practitioners, researchers, and donors highlights the need for a practitioner-friendly behavior model that links social norms to behavior. By allowing empirical exploration of the pathways through which social norms influence behavior, such a model would allow evidence to be generated, systematically, on the relationship between social norms and behavior. Our study finds that the FBM is an extremely useful model for exploring the pathways through which social norms affect behavior.

Our analysis highlights the important effects of motivation and ability on behavior. It illustrates the need to explore what effects social norms have on behavior using a behavior model that allows multiple pathways of effects to be considered. Researchers and intervention designers should be careful and not assume that social norms influence behavior through motivation. They should t consider the possibility that social norms may influence behavior though another viable pathway, ability.

## Data Availability

The dataset used for the study is available from the corresponding author on reasonable request.
